# Mutagenesis by host antimicrobial peptides: insights into microbial evolution during chronic infections

**DOI:** 10.15698/mic2014.07.157

**Published:** 2014-06-29

**Authors:** Dominique H. Limoli, Daniel J. Wozniak

**Affiliations:** 1 Department of Microbial Infection and Immunity, Ohio State University, Columbus, Ohio, USA.

**Keywords:** mutagenesis, antimicrobial peptides, cystic fibrosis, Pseudomonas aeruginosa, DNA repair

## Abstract

Antimicrobial peptides (AMPs) are produced by the mammalian immune system to fight invading pathogens. The best understood function of AMPs is to integrate into the membranes of microbes, thereby disrupting and killing cells. However, a recent study [*PLoS Pathogens* (2014) 10, e1004083] provides evidence that at subinhibitory levels, AMPs promote mutations in bacterial DNA, which enhance bacterial survival. In particular, in the bacterium *Pseudomonas aeruginosa,* one AMP called LL-37 can promote mutations, which enable the bacteria to overproduce a protective sugar coating, a process called mucoid conversion. *P. aeruginosa* mucoid conversion is a major risk factor for those suffering from cystic fibrosis (CF), one of the most common lethal, heritable diseases in the US. LL-37 was found to produce mutations by penetrating the bacterial cell and binding to bacterial DNA. It was proposed that LL-37 binding DNA disrupts normal DNA replication and potentiates mutations. Importantly, LL-37 induced mutagenesis was also found to promote resistance to rifampicin in both *P. aeruginosa* and *E. coli. *This suggests that AMP-induced mutagenesis may be important for a broad range of chronic diseases and pathogens.

Cystic fibrosis (CF) is a devastating inherited disease where patients currently suffer from the day they are born through the duration of their lives. Research over the past few decades has significantly improved patient outcome and quality of life. However, the current life expectancy is still only 38 years of age and patients persistently struggle with chronic pulmonary infections. Nearly all CF patients become colonized with the bacterium *Pseudomonas aeruginosa*, which becomes almost impossible to eradicate, particularly once converted to the mucoid phenotype. Mucoid conversion is characterized by the overproduction of the polysaccharide alginate and results from acquisition of stable mutations in alginate regulatory genes. Alginate overproduction confers a selective advantage for *P. aeruginosa *in the CF lung by providing recalcitrance to currently available therapeutics and host antimicrobials. Despite extensive investigation of mucoid *P. aeruginosa* over the past six decades, effective methods to prevent mucoid conversion or to eradicate mucoid *P. aeruginosa* remain elusive.

In-depth genetic analyses of *P. aeruginosa* factors controlling alginate overproduction have significantly advanced understanding of mucoid conversion. However, it remains to be understood why mucoid conversion is so prevalent in CF patients and if this process can be prevented. In the current study, we developed a selection strategy to isolate mucoid colonies in the laboratory and were able to interrogate which CF host factors are capable of promoting mucoid conversion. Initial investigations focused on polymorphonucleocytes (PMNs) and reactive oxygen species (ROS), as these host factors are enriched in the CF pulmonary environment and have previously been shown to induce mucoid conversion. Interestingly, while we observed both PMNs and ROS promote mucoid conversion, PMNs are still capable of inducing mucoid conversion in the absence of an oxidative burst response. These observations directed our focus towards non-oxidative PMN factors and we observed that at sub inhibitory levels, the cationic antimicrobial peptide LL-37 is capable of promoting mutations within the gene encoding the primary negative regulator of mucoid conversion, *mucA.* Importantly, the LL-37-induced mutations within *mucA* were found to be similar to the spectrum of mutations observed in mucoid isolates from CF patients. To further investigate the relevance of mutagenesis by LL-37 during infection we immune-depleted LL-37 from sputum derived from CF patients and determined that LL-37 directly contributes to mucoid conversion in these samples. Moreover, we observed that alginate-overproducing *P. aeruginosa* are more resistant to killing by lethal levels of LL-37 compared to the non-mucoid parental strains, uncovering an additional mechanism by which mucoid variants may be selected for during infection. Together, these data provide evidence that LL-37 may contribute to mutagenesis and pathoadaptation of *P. aeruginosa* in the CF pulmonary environment.

Intrigued by the observation that a host protein, whose primary function is to disrupt bacterial membranes and lyse cells, could promote mutagenesis, we sought to interrogate the mechanism of LL-37 induced mutagenesis. First, we examined if LL-37 induced mutagenesis is specific to the *mucA* gene and/or exclusive to *P. aeruginosa.* LL-37 was found to promote rifampin resistance in both *P. aeruginosa* and *E. coli*, suggesting that LL-37 may function as a general bacterial mutagen. Mechanistic studies began by examining the contribution of the translesion DNA polymerase DinB, which, by participating in error-prone translesion DNA synthesis, is critical for ROS-induced *mucA* mutagenesis. Importantly, DinB was also found to be required for LL-37 induced mutagenesis. We initially hypothesized that LL-37 may induce *dinB* by upregulation of membrane stress and/or SOS response pathways (including *dinB*). However, we did not find evidence for altered regulation of either pathway. Perplexed by these results we immune-labeled LL-37 and visualized LL-37-treated *P. aeruginosa* microscopically. We observed that LL-37 passed through the bacterial cell envelope, gained access to the cytosol and interacted with bacterial DNA. To test the hypothesis that LL-37/DNA interactions are important for mutagenesis, we generated DNA-binding deficient LL-37 variants, which abolished the ability of LL-37 to promote mutagenesis. Together these data suggest that at sub inhibitory levels, LL-37 promotes bacterial mutagenesis by passing through the cell envelope, without inducing significant membrane stress, and interacting with bacterial DNA. We propose that LL-37/DNA interactions promote error-prone DNA replication by DinB, which generates the mutations (see Figure 1 for model).

**Figure 1:**
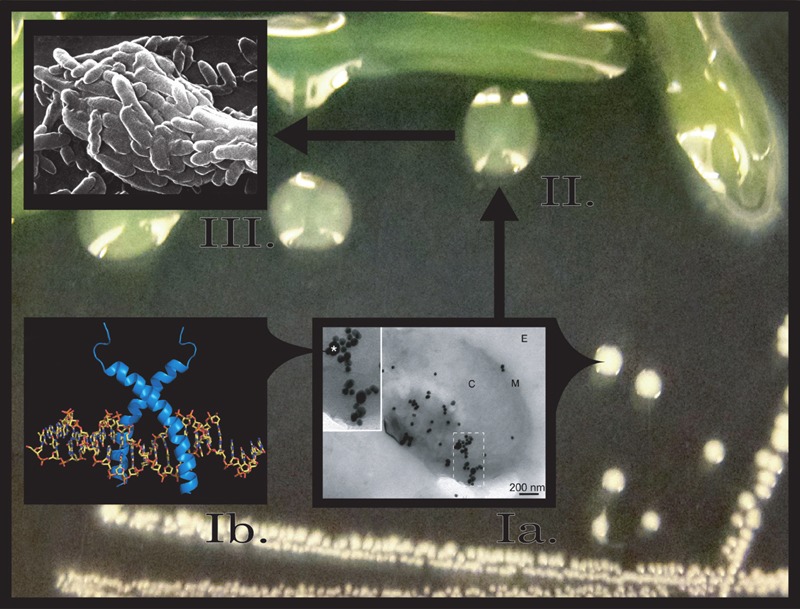
FIGURE 1: Proposed model of LL-37 induced mutagenesis and mucoid conversion*.* Step Ia, at low doses LL-37 (*) interacts with *Pseudomonas aeruginosa *and enters bacterial cells, binding to DNA (Step Ib). LL-37/DNA interactions promote *mucA* mutagenesis and conversion to the mucoid phenotype (Step II). Mucoid *P. aeruginosa* biofilms are now more resistant to killing by lethal levels of LL-37 and are selected for in the CF pulmonary environment (Step III).

Several studies are underway in our laboratory to further define the mechanism of LL-37 induced mucoid conversion and mutagenesis. We are currently defining how LL-37/DNA interactions induce DinB. Typical translesion DNA synthesis occurs when the replisome stalls upon encountering damaged DNA or a challenging template and low-fidelity polymerases like DinB will displace Pol III in order to perpetuate replication. Since LL-37 interactions with DNA are required for LL-37-induced mutagenesis, we postulate that LL-37 presents a physical barrier that stalls Pol III, inducing a switch to DinB, whose error-prone replication promotes mutagenesis. DNA binding assays suggest that LL-37 non-specifically interacts with DNA; however, peptides have been identified which specifically interact with DNA repair intermediates, such as Holliday junctions. Therefore, an alternative hypothesis could be that LL-37 perturbs effective DNA repair by binding to repair intermediates. In this respect, we are also working to define the DNA interactions and affinity of LL-37 in terms of DNA sequence and structure.

These data present a novel function for a host derived antimicrobial peptide and lend insight into mechanisms of bacterial persistence during chronic infection. Chronic pulmonary infections in CF patients produce significant inflammation and persistent influx of PMNs to the lungs, yet bacterial infections persist for decades. While the damaging effects of chronic hyper inflammation to surrounding host tissues has long been investigated, the impact on the bacterial communities is largely uncharacterized. Evidence from this study and others suggest one explanation for inefficient bacterial clearance in chronic infections may be sub inhibitory availability of host antimicrobial peptides. While total levels of host cationic peptides are elevated during infection, they may be inactive or sequestered, unable to sufficiently kill invading pathogens. This study provides evidence to suggest that these sub inhibitory levels may instead promote bacterial mutagenesis and pathoadaptation during chronic infection, whereby the immune response is inadvertently promoting chronic bacterial infection. These data highlight the importance of evaluating the impact of host-derived molecules and current and novel therapeutics on chronic bacterial communities.

